# Resting-state gamma power in schizophrenia: a systematic review and meta-analysis

**DOI:** 10.3389/fpsyt.2025.1731645

**Published:** 2026-01-19

**Authors:** Yong Liu, Pingping Xu, Shaohua Hu

**Affiliations:** 1 Department of Psychiatry, The First Affiliated Hospital, Zhejiang University School of Medicine, Hangzhou, China; 2 The Zhejiang Key Laboratory of Precision Psychiatry, Hangzhou, China; 3 Department of Psychiatry, The Third Affiliated Hospital of Zhejiang Chinese Medical University, Hangzhou, China; 4 Nanhu Brain-Computer Interface Institute, Hangzhou, China; 5 MOE Frontier Science Center for Brain Science and Brain-Machine Integration, Zhejiang University School of Medicine, Hangzhou, China; 6 Brain Research Institute of Zhejiang University, Hangzhou, China; 7 The State Key Lab of Brain-Machine Intelligence, Zhejiang University, Hangzhou, China; 8 Zhejiang Engineering Center for Mathematical Mental Health, Hangzhou, China

**Keywords:** cognition, EEG, gamma-band oscillations, resting state, schizophrenia

## Abstract

Gamma-band oscillations, generated by excitatory-inhibitory circuit interactions, are strongly implicated in schizophrenia, yet evidence on resting-state abnormalities remains inconsistent. We conducted a systematic review and meta-analysis of EEG and MEG studies comparing resting-state gamma activity in patients with schizophrenia and healthy controls, following PRISMA guidelines and assessing study quality with the Newcastle-Ottawa Scale. Twenty studies (n = 998 patients; n = 952 controls) were included. Standardized mean differences (Hedges’ g) were calculated and pooled using random-effects models. Results demonstrated a significant elevation of whole-brain gamma power in schizophrenia (g=0.371; 95% CI = 0.119–0.622; *P* < 0.001; I² = 78.2%). Region-specific analyses showed increases in frontal and temporal cortices, with smaller or inconsistent effects in parietal, occipital, and default mode network (DMN) regions. Meta-regression revealed illness duration (β=1.13) and medication status (β=0.43) as positive predictors, while eyes-open resting conditions attenuated effects (β=−0.70), indicating that both clinical chronicity and methodological factors contribute to heterogeneity. Publication bias was not evident by Egger’s test, although trim-and-fill suggested five potentially missing small-effect studies, reducing the pooled estimate to g=0.130. Sensitivity analyses confirmed that findings were not driven by outliers, and GRADE assessments rated the certainty of evidence as moderate for whole-brain gamma and low for regional outcomes. Taken together, these findings suggest that resting-state gamma power differences in schizophrenia represent a small and heterogeneous group-level effect, shaped by illness duration, medication status, and recording conditions. Rather than indicating a uniform abnormality, the results underscore substantial variability across studies and highlight the need for cautious interpretation. Future large-scale, longitudinal, and multimodal investigations-particularly in unmedicated and first-episode patients-are warranted to clarify the temporal dynamics, causal mechanisms, and potential translational relevance of resting-state gamma activity in schizophrenia.

## 1 Introduction

Schizophrenia is a chronic, severe psychiatric disorder with a lifetime prevalence of approximately 1% worldwide (1) and about 0.7% in China (2). Clinically, it presents with a heterogeneous constellation of symptoms, including positive symptoms (e.g., delusions, hallucinations), disorganized thought and behavior, and negative symptoms such as anhedonia and affective blunting (3). These features contribute to profound functional disability and excess mortality (4, 5), rendering schizophrenia one of the leading contributors to the global burden of disease (6).

Pathophysiologically, converging evidence implicates glutamatergic dysfunction as a core mechanism. The glutamatergic hypothesis posits hypofunction of N-methyl-D-aspartate (NMDA) receptors on parvalbumin-expressing γ-aminobutyric acid (GABA) interneurons (PV interneurons) as a pivotal abnormality. This deficit compromises inhibitory tone, disrupts the excitation-inhibition balance, and induces cortical hyperexcitability along with downstream dysregulation of dopaminergic mesolimbic and mesocortical pathways (7, 8). A neurophysiological signature of this imbalance is altered gamma-band oscillatory activity (30–100 Hz), which arises from the dynamic interaction of excitatory pyramidal neurons and inhibitory PV interneurons within the pyramidal-interneuron gamma (PING) circuit (9). The ~25 ms decay time of PV interneuron-mediated inhibition underlies the canonical 40 Hz rhythm, linking microcircuit dysfunction to aberrant gamma oscillations (10, 11).

Electroencephalography (EEG) and magnetoencephalography (MEG) enable direct, millisecond-resolution recordings of neuronal oscillations *in vivo*. MEG provides superior spatial resolution due to minimal field distortion, whereas EEG remains more widely used in both clinical and research contexts because of its accessibility. Together, the two modalities are complementary for characterizing cortical oscillatory dynamics (12). Gamma oscillations are of particular interest because they undergo marked developmental changes during adolescence – the peak age of schizophrenia onset (13) – and show abnormalities as early as the first episode of illness (14, 15). These features support the hypothesis that gamma oscillations constitute a translational biomarker of schizophrenia-related circuit dysfunction.

Nonetheless, the directionality of resting-state gamma abnormalities in schizophrenia remains unresolved. While some studies report elevated gamma power relative to healthy controls, others describe reductions or null findings (16, 17). Previous reviews have largely focused on task-related gamma alterations during perceptual or working-memory paradigms (18, 19), whereas findings on resting-state gamma remain fragmented and lack quantitative synthesis.

The present study addresses this gap by conducting a systematic review and meta-analysis of resting-state gamma activity in schizophrenia. Specifically, we aimed to (i) compare gamma-band power between patients and healthy controls, and (ii) explore associations with illness duration, and where available, symptom severity across core psychopathological domains. By integrating disparate findings, this work seeks to clarify whether resting-state gamma abnormalities represent a robust electrophysiological feature of schizophrenia or are modulated by secondary, context-dependent factors.

## 2 Methods

### 2.1 Search strategy and study selection

The search strategy and study selection processes were performed in accordance with the PRISMA guidelines (20). We systematically searched for relevant observational studies comparing individuals with schizophrenia and healthy controls in the following databases: MEDLINE (via PubMed), EMBASE, PsycINFO (via EBSCO), CINAHL (via EBSCO), LILACS, the Cochrane Library, the Chinese National Knowledge Infrastructure (CNKI), the Chongqing VIP Database for Chinese Technical Periodicals (CQVIP), WANFANG DATA, and the Chinese Biomedical Literature Database (CBM), including studies published before September 1, 2025. The following key words were used: (“EEG” OR “electroencephalography” OR “electroencephalogram” OR “MEG” OR “magnetoencephalography”) AND “resting-state” AND (“schizophrenia” OR “first episode of psychosis”). The review was also conducted in Chinese using the following search terms: “精神分裂症” (schizophrenia), “健康对照” (healthy controls), “静息态” (resting state), “伽马波” or “γ波” (gamma band or γ-band), “脑电图” (EEG), and “脑磁图” (MEG). Boolean operators and equivalent synonyms were applied to ensure comprehensive retrieval across Chinese databases. The eligibility criteria and study objectives were defined *a priori* and structured according to the PICOS framework, including population (individuals with schizophrenia), exposure (resting-state EEG/MEG gamma activity), comparison (healthy controls), outcomes (gamma-band power measures), and study design (observational studies).

Identified articles were imported into EndNote X9, and duplicate records were removed. Two reviewers independently screened the titles and abstracts of the remaining articles to identify potentially eligible studies. Full-text articles were retrieved for further assessment when either reviewer deemed the study potentially eligible or expressed uncertainty about eligibility. Additionally, the reference lists of all full-text articles were manually searched to identify further relevant studies. The same two reviewers then independently assessed the full texts based on the predefined inclusion and exclusion criteria. Any discrepancies were resolved through discussion among all review authors until consensus was reached.

### 2.2 Inclusion and exclusion criteria

Eligible studies included observational (cross-sectional or case–control) investigations comparing individuals with schizophrenia, diagnosed according to standardized criteria (DSM, ICD, or CCMD), with healthy controls, and reporting quantitative measures of resting-state EEG or MEG gamma-band activity. Studies were excluded if they involved ultra-high-risk populations, animal, or *in vitro* models, lacked a healthy control group, did not provide extractable gamma-specific data, or were reviews, meta-analyses, case reports, conference abstracts, or duplicate/overlapping publications (in which case the most complete dataset was retained).

### 2.3 Data extraction and evaluation

Data from eligible studies were independently extracted by two reviewers using a standardized form, including study details (author, year, region, design, sample size), participant characteristics (diagnostic criteria, illness duration, demographics, medication status), EEG/MEG parameters (modality, channels, analysis level), definitions and measures of gamma activity (frequency range, absolute/relative power, amplitude, resting condition), and reported outcomes (means, variability indices, effect sizes, p-values). When necessary, quantitative data were obtained from published figures using WebPlotDigitizer (v4.6). Discrepancies between reviewers were resolved through discussion or adjudication by a third reviewer. When overlapping samples were identified across multiple reports, the study with the largest sample size or most complete dataset was retained.

The methodological quality of included studies was independently assessed using a modified Newcastle-Ottawa Scale (NOS) adapted for observational neuroimaging studies. The NOS evaluates three domains: (i) selection of study groups, (ii) comparability of groups, and (iii) ascertainment of exposure/outcome. Each study was assigned a quality rating (high, moderate, or low) based on total NOS scores, and sensitivity analyses were planned to examine the influence of study quality on pooled estimates.

### 2.4 Types of outcome measures

The primary outcome was whole brain resting-state gamma-band activity in schizophrenia relative to healthy controls. Extracted measures included spectral power indices (absolute, relative, log- or dB-scaled), amplitude-based indices (e.g., RMS), and gamma activity defined within 30–100 Hz or its sub-bands (30–50 Hz, 50–100 Hz). Outcomes were further stratified by recording condition (eyes open *vs*. eyes closed). Secondary outcomes included associations of gamma activity with illness duration and symptom severity (e.g., PANSS, SANS/SAPS). When multiple gamma measures were reported, all indices were extracted to enable subgroup and sensitivity analyses.

### 2.5 Statistical analysis

#### 2.5.1 Effect size calculation

For each study, standardized mean differences (Hedges’ g) and corresponding standard errors (SE) were calculated to quantify group differences in resting-state gamma-band activity between individuals with schizophrenia and healthy controls. When studies reported multiple sub-bands (e.g., low *vs*. high gamma), recording conditions (eyes open *vs*. eyes closed), or cortical regions, separate effect sizes were computed and, when appropriate, combined using fixed-effect models to obtain a single study-level estimate. For studies reporting medians and interquartile ranges, values were converted to means and standard deviations using established formulas. Given the greater consistency of reporting, analyses focused on the low gamma band (30–50 Hz), whereas insufficient data precluded meta-analyses of other gamma sub-bands. To ensure independence of effect sizes, one meta-analysis was conducted for whole-brain gamma activity, along with eight separate meta-analyses for the major cortical regions and DMN-related regions. In addition, secondary analyses were performed to examine the influence of potential outliers, defined as effect sizes more than two standard deviations from the overall mean.

#### 2.5.2 Meta-analysis model, heterogeneity, publication bias, and sensitivity analyses

Pooled effect sizes were estimated using a random-effects model with restricted maximum likelihood (REML), interpreting Hedges’ g values of 0.2, 0.5, and 0.8 as small, moderate, and large, respectively. Between-study heterogeneity was assessed with Cochran’s Q test and quantified using the I² statistic (25%, 50%, and 75% representing low, moderate, and high heterogeneity), and prediction intervals were calculated to estimate the range of true effects. Potential publication bias was examined through funnel plots, Egger’s regression, and Begg’s rank correlation tests; when asymmetry was indicated, the trim-and-fill procedure was applied to adjust pooled estimates. Sensitivity analyses were performed by iteratively excluding individual studies (leave-one-out approach) and by restricting analyses to high-quality studies (based on NOS ratings) to evaluate the robustness of findings.

#### 2.5.3 Subgroup and meta-regression analyses

To explore potential sources of heterogeneity in whole-brain gamma power, we conducted meta-regression analyses using linear mixed-effects models (LMMs) implemented in Python. Four study-level variables were selected *a priori* as fixed effects based on their theoretical relevance and data availability: (i) mean patient age, given the established age-related changes in neural oscillations; (ii) medication status (categorized as medicated, unmedicated/drug-free, or mixed [patients receiving both antipsychotics and unmedicated/drug-free subgroups]), due to the known influence of antipsychotics on gamma activity; (iii) illness duration (in months), reflecting potential progression effects across disease chronicity; and (iv) resting-state condition (eyes open *vs*. eyes closed), which is known to modulate background oscillatory dynamics. Study ID was modeled as a random intercept to account for clustering of effect sizes within studies, and effect sizes were weighted by the inverse of their variance.

All analyses were performed in Python 3.11 within the PyCharm (JetBrains) IDE. A two-tailed p-value < 0.05 was considered statistically significant.

## 3 Results

### 3.1 Study selection

**Figure 1:**
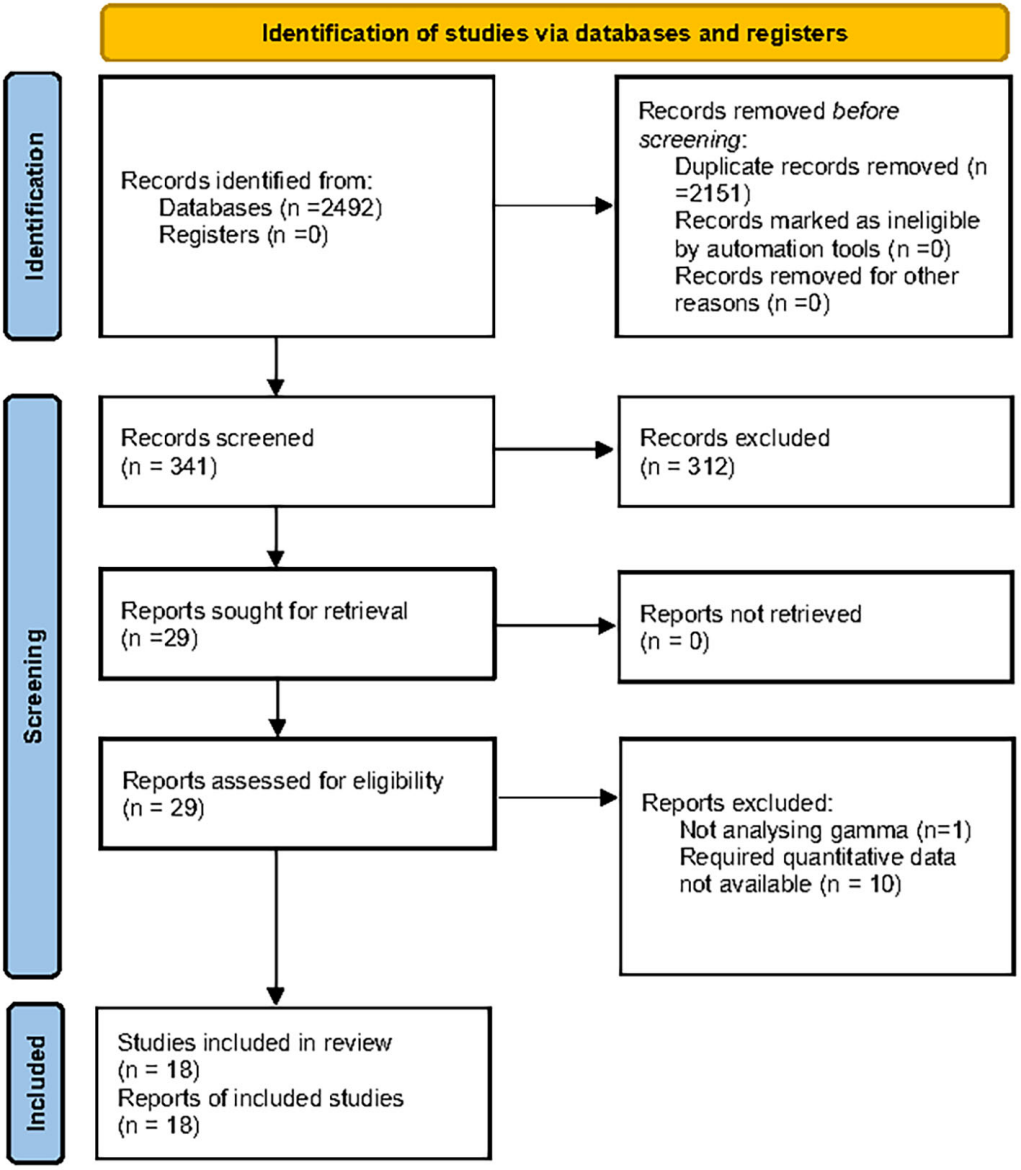
PRISMA 2020 flowchart of study selection.

The flow of study selection is shown in **Figure 1**. The initial search yielded a total of 449 records, and 29 full-text articles were assessed for eligibility. Out of the full-text articles, 11 were excluded due to irrelevant outcomes, intervention, study design, or no data were available. Finally, 18 full-text articles (16, 17, 21–37) fulfilled the eligibility criteria for the systematic review and meta-analysis.

### 3.2 Study characteristics

18 studies were included in the systematic review and meta-analysis, comprising a total of 998 patients with schizophrenia (mean age: 38.8 ± 12.4 years) and 952 healthy controls (mean age: 36.0 ± 17.2 years). 16 studies employed EEG and two used MEG to assess resting-state gamma-band activity. Sample sizes varied considerably, from small cohorts of 15 patients and 15 controls (28) to large samples exceeding 150 patients (34). The included populations ranged from first-episode, unmedicated patients (e.g., 30, 31, 36) to chronic medicated cohorts with long illness duration (e.g., 27, 35). Gamma frequency ranges analyzed were generally 30–50 Hz, with extensions to 30–70 Hz or 30–100 Hz in some studies. Most investigations reported sensor-level data, while several performed source-level analyses (e.g., 25, 30, 35). Brain regions analyzed varied across studies, including whole-brain gamma power (e.g., 27, 37), lobe-level regions (frontal, parietal, temporal, occipital, midline), and default mode network (DMN) subregions (mPFC, PCC). Medication status was heterogeneous: some cohorts were unmedicated or drug-naïve, others were chronically treated with antipsychotics at chlorpromazine-equivalent doses ranging from ~200 to >500 mg/day, and some included mixed medicated/unmedicated samples. Overall, the included studies represent a diverse set of clinical populations and methodological approaches, providing a comprehensive dataset for meta-analysis of resting-state gamma-band abnormalities in schizophrenia. Selected characteristics of the included studies are shown in **Table 1**.

**Table 1:** Characteristics of the included studies.

Author	EEG/MEG	Gamma frequency	Diagnosis	Eyes	Regions	No.		Mean age		Duration (months)	Medication status	Image analysis level
						SZ	HC	SZ	HC			
Rutter et al., 2009 (21)	275-MEG	30–80Hz	Sz	closed	bilateral precuneus, cuneus, PCC	38	38	31.2 ± 9.8	32.5 ± 10.8	Na	Na	Source
J.W.Y. Kam et al., 2013 (23)	32-EEG	30–50Hz	Sz	closed	Frontal, Central, Parietal, Temporal	132	136	40	39	Na	Mixed	Sensor
S.K. Tikka et al., 2013 (24)	192-EEG	30–50Hz	Sz	closed	Frontal, Parietal, Temporal, Occipital, Midline	20	20	29.6 ± 8.3	27.5 ± 5.4	52.2 ± 44.9	Unmedicated	Sensor
Kim et al., 2014 (25)	306-MEG	30–50Hz	Sz	opened	L mPFC, L PCC	20	20	22.8 ± 3.9	22.1 ± 2.0	70 ± 36	Antipsychotics	Source
S. Mitra et al., 2015 (28)	192-EEG	30–50Hz	Sz	closed	Frontal, Parietal, Temporal, Occipital, Midline	15	15	28.9 ± 6.8	29.4 ± 5.9	55.13	Antipsychotics	Sensor
A. Ramyead et al., 2015 (30)	19-EEG	30–50Hz	FES	closed	L MFG	31	29	30.8 ± 8.9	22.4 ± 5.0	Na	Unmedicated	Source
S.K. Tikka et al., 2015 (29)	192-EEG	30–50Hz	Sz	closed	Frontal, Parietal, Temporal, Occipital, Midline	30	30	29.9 ± 8.3	28.1 ± 7.0	63.2 ± 6.31	Unmedicated	Sensor
Y. Hirano et al., 2015 (27)	71-EEG	30–100Hz	Sz	Unknown	Whole Brain	18	18	45.4 ± 8.9	44.1 ± 6.7	234 ± 114	Mixed	Source
Z. Garakh et al., 2015 (26)	19-EEG	30–40Hz	Sz	closed	Left/Right Frontal	32	40	28.91 ± 10.64	27.6 ± 6.4	Na	Mixed	Sensor
S. Umesh et al., 2018 (16)	192-EEG	30–50Hz	Sz	closed	Frontal, Parietal, Temporal, Occipital, Midline	20	20	29.8 ± 7.7	29.8 ± 7.7	Na	Antipsychotics	Sensor
Arikan et al., 2018 (31)	19-EEG	30–50Hz	Sz	closed	Left frontal (F3), Left parietal (C3), Midline (Cz)	23	11	39	37.5	>6	Unmedicated	Sensor
Tanaka-Koshiyama et al., 2020 (34)	40-EEG	30–50Hz	Sz	opened	Whole Brain	157	145	46.4 ± 10.8	39.9 ± 12.8	300 ± 144	Antipsychotics	Sensor
D. Fresche et al., 2020 (32)	64-EEG	30–45Hz	Sz	opened	Frontal, Parietal, Temporal, Occipital, Midline	21	27	40 ± 8	35 ± 11	232 ± 96	Antipsychotics	Sensor
S. Kim et al., 2020 (33)	62-EEG	30–55Hz	Sz	closed	Whole Brain	38	30	43.2 ± 11.2	43.0 ± 12.4	145 ± 90.6	Mixed	Sensor
S. Yadav et al., 2021 (36)	192-EEG	31–50Hz	SSD	closed	Frontal, Parietal, Temporal, Occipital, Midline	29	30	25.8 ± 4.64	27.3 ± 6.0	13.3 ± 6.75	Unmedicated	Sensor
D. Koshiyama et al., 2021 (35)	40-EEG	30–50Hz	Sz	closed	R PCC	148	143	46.2 ± 10.9	39.6 ± 13.0	305 ± 142	Mixed	Source
Dario Gordillo et al., 2023 (37)	64-EEG	30–70 Hz	Sz	closed	Whole Brain	121	75	35.8 ± 9.2	35.1 ± 7.7	129.6 ± 104.4	Mixed	Sensor
M.S. Jacob et al., 2023 (17)	32-EEG	30–50Hz	Sz	opened	Left frontal (Fc5)	57	46	35.6 ± 13.9	38.3 ± 15.1	Na	Mixed	Sensor

Mixed, Antipsychotics+ Drug-free; Unmedicated, Drug-free+ Drug-naive; FES, First episode of schizophrenia; SSD, Schizophrenia spectrum disorders;SZ, Schizophrenia.

### 3.3 Risk of bias in included studies

Risk of bias was evaluated for all 18 included studies using a modified Newcastle–Ottawa Scale (NOS; maximum score = 9). Overall, 10 studies were judged as low risk of bias (NOS = 8/9), reflecting strengths such as standardized diagnostic procedures (DSM-IV/ICD-10 SCID interviews), inclusion of drug-naïve or drug-free cohorts in several studies, use of high-density EEG/MEG with rigorous preprocessing pipelines, and appropriate statistical analyses with multiple-comparison correction. In contrast, 8 studies were rated as moderate risk of bias (NOS = 7/9), largely due to one or more of the following: group imbalances in age, sex, or education; lack of adjustment for chronic medication exposure and illness duration; or absence of explicit blinding during artifact rejection and data analysis. Importantly, no study was rated as high risk of bias (≤6/9), indicating that the overall methodological quality of the included literature was acceptable. A domain-level summary is illustrated in **Supplementary Figure S1** (traffic-light plot).

### 3.4 Results of syntheses

#### 3.4.1 Meta-analysis of whole-brain gamma power

##### 3.4.1.1 Characteristics and risk of bias

A total of 11 studies (n = 998 SZ; n = 952 HC) contributed to the whole-brain gamma power synthesis. Most were EEG-based, with sample sizes ranging from 15 to 157. Risk of bias assessment rated 7 studies as low risk and 4 as moderate risk (**Supplementary Figure S1**).

Effect size calculations revealed a range of Hedges’ g values from −0.18 to 0.84 across individual studies. Two studies reported negative effects, indicating reduced gamma power in patients with schizophrenia relative to healthy controls (g=−0.17 for spectral power in Kam et al. (23); g=−0.18 for spectral power in Umesh et al. (16)). Neither of these effects reached statistical significance. The remaining studies reported positive effect sizes, reflecting increased gamma power in schizophrenia.

##### 3.4.1.2 Statistical syntheses

**Figure 2:**
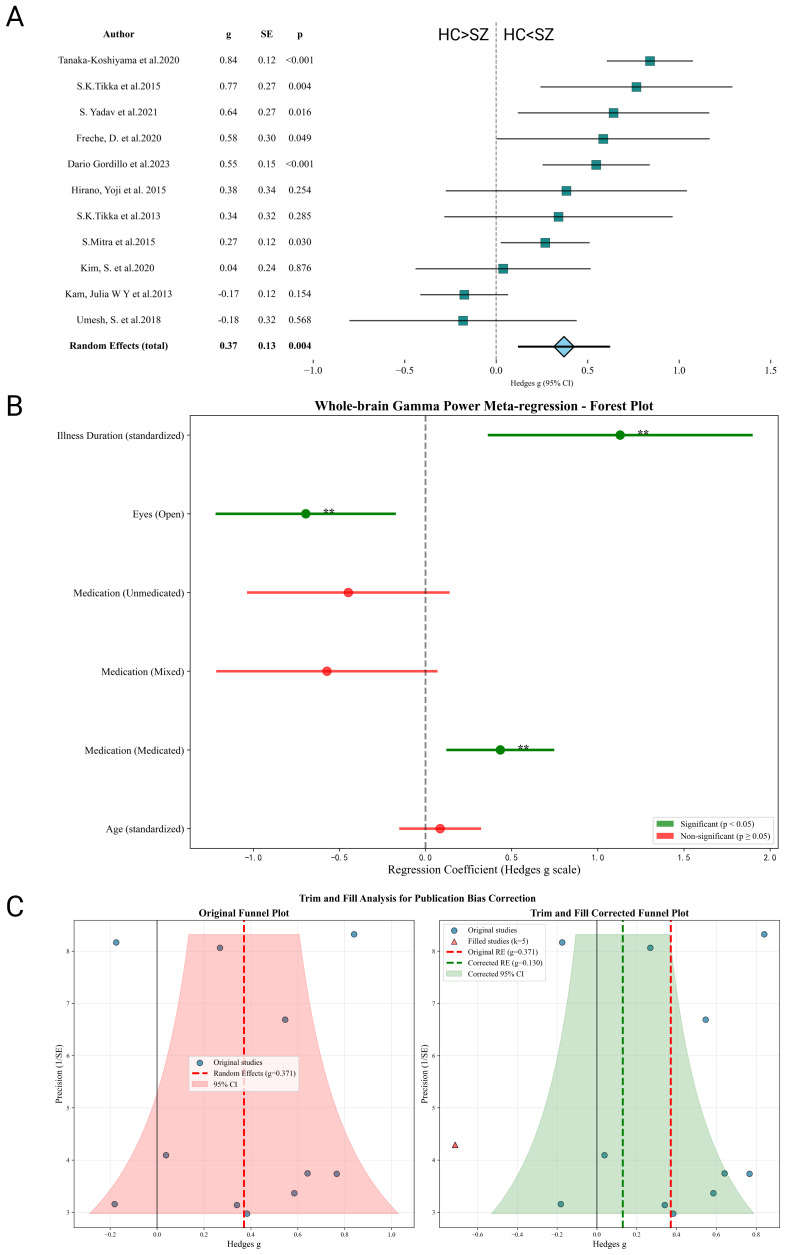
Whole-brain meta-analysis of resting-state gamma power in schizophrenia. **(A)** Forest plot showing a significant increase in whole-brain gamma power in patients (Hedges’ g = 0.371; 95% CI = 0.119–0.622; P = 0.004). No outliers were detected. **(B)** Meta-regression results: illness duration (β=1.13) and medication status (β =0.43) predicted stronger effects, whereas eyes-open condition attenuated effects (β=–0.70). **(C)** Funnel plot: no strong evidence of publication bias (Egger’s test P = 0.307), though trim-and-fill suggested five missing small-effect studies, reducing the pooled effect to g=0.130.

The pooled meta-analysis demonstrated that, compared with healthy controls, patients with schizophrenia exhibited significantly elevated whole-brain gamma power (Hedges’ g=0.371; 95% CI = 0.119–0.622; P = 0.004) (**Figure 2A**). This corresponds to a moderate effect size. Initial diagnostic checks indicated that none of the study-level effect sizes deviated by more than two standard deviations from the pooled mean (mean g=0.371, SD = 0.317). Accordingly, no studies were treated as outliers, and the random-effects estimate remained unchanged at g=0.371, underscoring the robustness of the finding.

##### 3.4.1.3 Causes of heterogeneity

However, significant between-study heterogeneity was observed (τ² = 0.128; Q = 45.79, df=10, P<0.001; I² = 78.2%), suggesting substantial variability across individual studies. Meta-regression identified several moderators contributing to the observed heterogeneity. Illness duration showed the strongest positive association with whole-brain gamma power (β =1.13, *p* < 0.01), indicating that patients with longer illness duration exhibited more pronounced gamma abnormalities. Medication status also emerged as a significant positive predictor (β = 0.43, p < 0.01). Importantly, medication status was coded as a study-level categorical variable (Antipsychotics, Unmedicated, or Mixed) based on information reported in the original studies, and did not capture medication dose, duration of exposure, or specific antipsychotic classes. Accordingly, this association should be interpreted cautiously and may reflect differences in sample composition or illness chronicity across studies rather than a direct pharmacological effect of antipsychotic treatment on gamma power. By contrast, resting-state condition exerted a negative effect: eyes-open recordings were associated with reduced gamma power differences relative to eyes-closed (β = −0.70, *p* < 0.01), consistent with the role of visual input and arousal in modulating oscillatory activity. Age and the mixed-medication subgroup were not significant predictors.

In summary, illness chronicity appears to be the strongest contributor to gamma power abnormalities, medication exerts a moderate positive effect, whereas eyes-open recordings attenuate group differences. Together, these moderators account for part of the substantial between-study heterogeneity (I² =78.2%) (**Figure 2B**).

##### 3.4.1.4 Sensitivity analyses

A series of sensitivity analyses were conducted to evaluate the robustness of the meta-analytic findings. Leave-one-out analyses were performed to assess whether the overall estimate was driven by any single study. The pooled random-effects estimate remained stable across study omissions, with pooled Hedges’ g ranging from 0.303 to 0.454, indicating that no individual study exerted a disproportionate influence on the overall result. Outlier diagnostics indicated no influential studies. The prespecified outlier range (Hedges’ g = −0.537 to 1.303) did not identify any effect sizes outside this interval, and exclusion of potential outliers did not alter the pooled estimate (Hedges’ g = 0.371 before and after exclusion), suggesting that the overall result was not driven by extreme values. Exploratory subgroup sensitivity analyses were considered; however, several subgroups contained too few studies to permit reliable pooled estimation or formal between-group comparisons. Accordingly, subgroup analyses were not formally conducted. Taken together, these findings suggest that the observed increase in resting-state gamma power in schizophrenia is not attributable to single influential studies or extreme values, although further sensitivity analyses incorporating more detailed and consistently reported study-level data would be valuable.

##### 3.4.1.5 Reporting biases

The funnel plot of the 11 included studies is shown in **Figure 2C**. Visual inspection revealed a relatively symmetrical distribution of effect sizes, and Egger’s regression test was non-significant (intercept=0.130; *P* = 0.307), indicating no strong evidence of small-study effects or publication bias. However, application of Duval and Tweedie’s “trim-and-fill” method suggested that up to five studies may be missing, likely representing unpublished or unreported small-effect studies. After imputing these studies, the overall pooled effect size decreased from Hedges’ g=0.364 to g=0.130, suggesting that the observed moderate effect may be somewhat overestimated due to potential reporting bias.

#### 3.4.2 Meta-analysis of other brain regions gamma power

##### 3.4.2.1 Meta-analysis of regional gamma power

**Figure 3:**
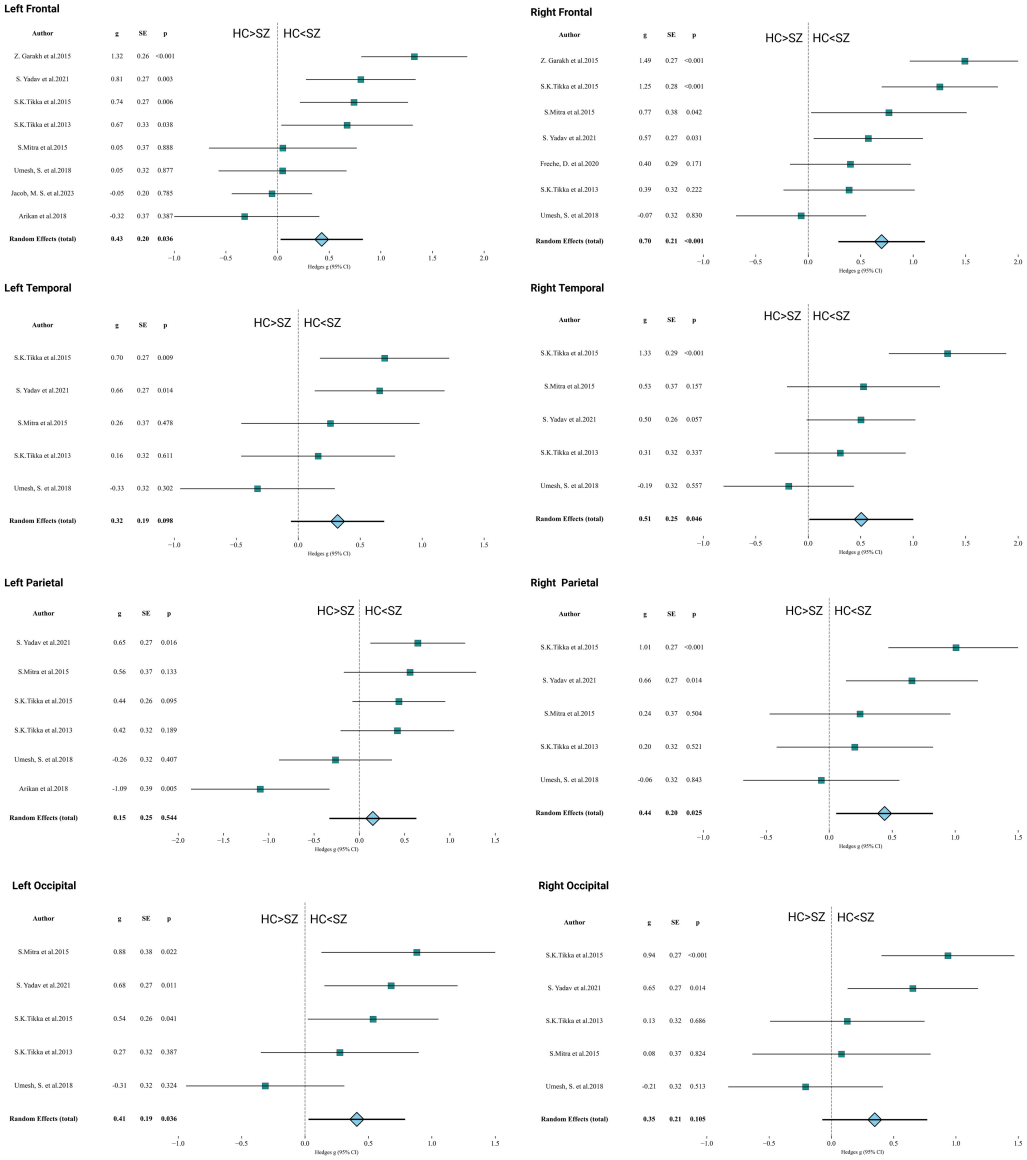
Regional analyses of resting-state gamma power. Regional analyses: significant increases in left frontal (g=0.428), right frontal (g=0.699), right temporal (g=0.505), right parietal (g=0.439), and left occipital (g=0.408) cortices; other regions showed non-significant trends.

Beyond whole-brain analyses, several studies reported regional gamma power: Left frontal cortex (8 studies): Hedges’ g = 0.428; 95% CI = 0.029–0.826; P = 0.036.Right frontal cortex (7 studies): Hedges’ g = 0.699; 95% CI = 0.287–1.112; P < 0.001.Left temporal cortex (5 studies): Hedges’ g = 0.318; 95% CI = –0.058–0.694; P = 0.098 (ns).Right temporal cortex (5 studies): Hedges’ g = 0.505; 95% CI = 0.010–1.000; P = 0.046.Left parietal cortex (6 studies): Hedges’ g = 0.149; 95% CI = –0.332–0.629; P = 0.544 (ns).Right parietal cortex (5 studies): Hedges’ g = 0.439; 95% CI = 0.055–0.824; P = 0.025.Left occipital cortex (5 studies): Hedges’ g = 0.408; 95% CI = 0.027–0.790; P = 0.036.Right occipital cortex (5 studies): Hedges’ g = 0.348; 95% CI = –0.073–0.769; P = 0.105 (ns). Overall pattern. Effects were largest and most consistent in the frontal cortices-particularly the right frontal region-while temporal and parietal effects were smaller and more variable; occipital findings were mixed, with significance in the left but not the right hemisphere (**Figure 3**).

##### 3.4.2.2 Meta-analysis of DMN regions gamma power

**Figure 4:**
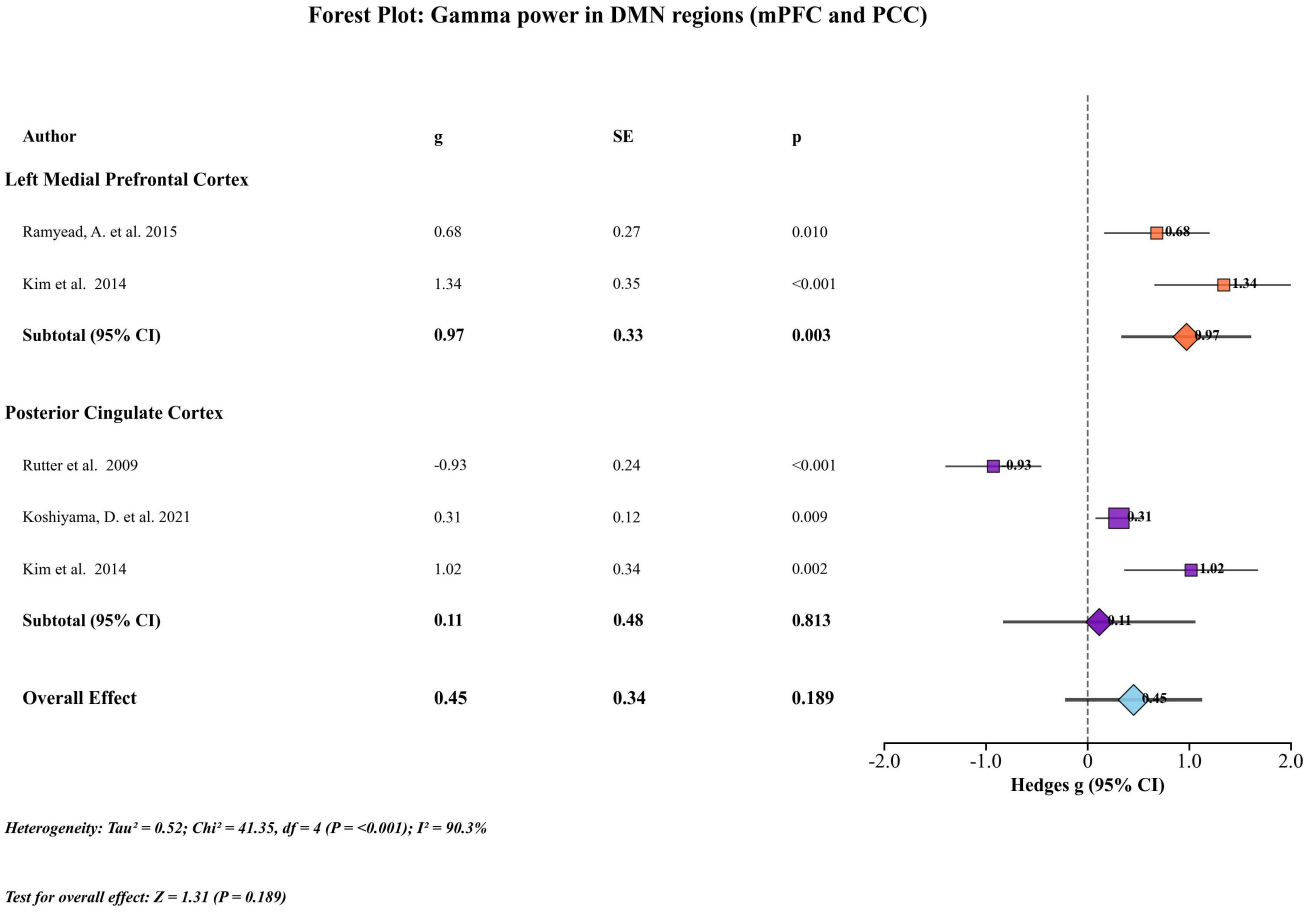
DMN subgroup analyses of resting-state gamma power. DMN analyses: pooled mPFC studies indicated elevated gamma power (g=0.970; 95% CI = 0.330–1.611; P = 0.003). PCC findings (EEG/MEG) also suggested abnormalities, though based on few studies.

Four studies provided data from default mode network (DMN)-related regions, specifically the medial prefrontal cortex (mPFC) and posterior cingulate cortex (PCC). Data were derived either from EEG source reconstruction (eLORETA) or MEG recordings. For the mPFC, two studies contributed data. One study used 19-channel EEG with eLORETA source localization, reporting values for the left medial frontal gyrus (BA9), while another used 306-channel MEG, reporting the left medial prefrontal cortex. The pooled analysis indicated a significant increase in gamma power in schizophrenia (Hedges’ g=0.970; 95% CI = 0.330–1.611; Z = 2.97; P = 0.003; I² = 55.5%). For the PCC, three studies contributed data. These included one 40-channel EEG source reconstruction reporting the right PCC, one 275-channel MEG study reporting bilateral precuneus, cuneus, and PCC activity, and one 306-channel MEG study reporting the left PCC. The pooled analysis showed a non-significant effect (Hedges’ g =0.114; 95% CI = −0.832–1.061; Z = 0.24; P = 0.813; I² = 93.0%). For the overall DMN effect, when pooling across all DMN-related nodes, patients with schizophrenia exhibited a moderate, non-significant increase in gamma power (Hedges’ g = 0.452; 95% CI = –0.222–1.127; Z = 1.31; P = 0.189; I² = 90.3%). (**Figure 4**).

##### 3.4.2.3 Certainty of evidence

The certainty of evidence for the primary outcome (resting-state whole-brain gamma power differences between patients with schizophrenia and healthy controls) was rated as moderate using the GRADE approach. Although most included studies were of low to moderate risk of bias, the overall certainty was downgraded due to substantial statistical heterogeneity (I² = 78.2%) and evidence of possible reporting bias suggested by the trim-and-fill analysis. The effect estimate was relatively precise (95% CI not crossing zero in the primary analysis), but the presence of potential unpublished small-effect studies and inconsistency across subgroups reduced confidence in the robustness of the finding.

For subgroup outcomes (regional gamma power, DMN-related regions), the certainty of evidence was judged as low, mainly due to the small number of contributing studies per region (n = 4–8), wide confidence intervals including the null in several cases, and greater susceptibility to reporting bias. Details are shown in **Supplementary Table S1**.

## 4 Discussion

This systematic review and meta-analysis provide quantitative evidence that patients with schizophrenia exhibit higher resting-state gamma power at the group level compared with healthy controls. The pooled effect for whole-brain gamma activity was statistically significant but accompanied by substantial heterogeneity, and region-specific analyses indicated increases in frontal and temporal cortices, with more limited or inconsistent findings in parietal, occipital, and DMN-related regions. Importantly, whole-brain gamma power estimates in this meta-analysis were derived from scalp/sensor-level EEG or MEG recordings, reflecting global oscillatory activity without source localization. By contrast, gamma abnormalities within DMN regions were based on a smaller subset of studies using source-reconstructed EEG or anatomically defined MEG estimates, and therefore should be interpreted with greater caution. Differences between sensor-level and source-level methodologies may partly contribute to the observed heterogeneity across studies. These findings are broadly consistent with prior task-related EEG/MEG literature highlighting disrupted gamma-band synchrony in schizophrenia (18, 38), and they extend existing knowledge by demonstrating that gamma alterations can also be observed during resting state.

Meta-regression analyses suggested that illness duration was the strongest positive predictor of gamma power differences, while medication status exerted a moderate positive association and eyes-open resting conditions attenuated group effects, indicating that both clinical chronicity and methodological factors contribute to between-study variability. Taken together, these results are compatible with models of impaired excitation-inhibition balance and pyramidal-interneuron circuit dysfunction in schizophrenia, as proposed by the glutamatergic hypothesis (39) and prior translational frameworks (11), while also underscoring the context-dependent and heterogeneous nature of gamma abnormalities across studies. A recent systematic review by De Pieri et al. (40) summarized a large body of resting-state EEG/MEG gamma findings in schizophrenia but included only four studies in their quantitative meta-analysis, yielding heterogeneous and non-significant pooled effects. In contrast, the present study synthesized a larger number of studies quantitatively and identified a significant elevation in whole-brain gamma power, albeit with substantial heterogeneity. Differences in the scope of quantitative synthesis and analytic strategies may partly explain these discrepant findings.

Several limitations of the available evidence should be acknowledged. This review was not prospectively registered in PROSPERO. However, the study protocol, including eligibility criteria and analytic strategy, was defined prior to data extraction and was not modified during the review process. Another important limitation concerns symptom severity. Although associations between resting-state gamma power and clinical symptoms were considered as a secondary objective, a quantitative synthesis was not feasible because symptom severity measures were inconsistently reported across studies, varied in scales and subdomains (e.g., PANSS, SANS/SAPS), and were available in only a small subset of the included literature. This gap highlights the need for future studies to systematically report standardized symptom measures alongside electrophysiological outcomes to better elucidate the clinical relevance of gamma-band abnormalities in schizophrenia. First, despite the overall moderate certainty rating by GRADE, substantial heterogeneity was observed (I²=78.2%), partly explained by clinical moderators but also reflecting methodological differences across studies. Second, many regional and DMN-specific analyses were based on only 4–8 studies each, with wide confidence intervals that often included the null, resulting in low certainty ratings. Third, most included studies enrolled medicated, chronic patients, limiting generalizability to unmedicated or first-episode populations (14, 15). Fourth, although Egger’s test suggested no strong publication bias, trim-and-fill analysis indicated that small-effect studies may be missing (41), implying that the pooled effect size may overestimate the magnitude of resting-state gamma differences.

The review process also has several limitations. Although we followed PRISMA guidelines (20, 42) and used a comprehensive multi-database search strategy, unpublished data, negative results, or non-English/Chinese studies may have been missed. Data extraction in some cases relied on digitization from figures (43), which may introduce minor measurement error. Our meta-regression analyses were limited by the number of available studies and incomplete reporting of important covariates, precluding a more extensive exploration of heterogeneity. Moreover, although the present meta-analysis focused on samples diagnosed with schizophrenia, the developmental trajectory of gamma-band abnormalities across different stages of psychosis remains an important unresolved question. Emerging evidence suggests that gamma oscillations may be differentially altered in early psychosis or first-episode populations, potentially reflecting dynamic changes in excitation-inhibition balance during illness progression (44). However, the limited number of early psychosis studies using comparable resting-state gamma measures currently precludes a quantitative synthesis. Future longitudinal studies spanning early psychosis to chronic stages will therefore be critical for determining whether resting-state gamma power abnormalities represent an early biomarker or a consequence of illness chronicity.

Despite these limitations, the present findings offer several important implications. Rather than indicating a uniform or large abnormality, the observed elevation of resting-state gamma power appears to represent a small and heterogeneous group-level effect, shaped by illness duration, medication status, and recording conditions. From a translational perspective, resting-state gamma measures may nonetheless provide complementary information about cortical circuit dysfunction in schizophrenia, particularly when interpreted in conjunction with clinical stage and methodological context.

In addition, an important distinction should be made between resting-state gamma activity and task-evoked or stimulation-induced gamma responses. Resting-state gamma power is thought to reflect baseline cortical excitability and tonic excitation-inhibition balance, whereas evoked gamma responses are more closely related to stimulus-driven synchronization and context-dependent network engagement (18, 45). Divergent directions of gamma abnormalities across resting and evoked conditions may therefore reflect state-dependent and mechanism-specific circuit dysfunction rather than contradictory findings, which may also contribute to the substantial heterogeneity observed across studies (38).

Clinically, gamma measures could complement existing neurophysiological assessments and may serve as a target for neuromodulatory interventions such as gamma-frequency transcranial alternating current stimulation (tACS) (46). At a policy level, these results highlight the need for standardized EEG/MEG protocols and transparent data sharing to reduce methodological heterogeneity and facilitate reproducibility (12). For future research, priority should be given to longitudinal studies including unmedicated and first-episode patients to disentangle illness progression from medication effects, as well as multimodal studies linking gamma abnormalities to neurochemical, structural, and cognitive outcomes. Large-scale collaborative datasets will be essential to increase precision, enable patient stratification, and assess the prognostic and therapeutic utility of resting-state gamma power in schizophrenia.

## Data Availability

The original contributions presented in the study are included in the article/**Supplementary Material**. Further inquiries can be directed to the corresponding author.

## Appendix. Search Strategies

MEDLINE (via PubMed): 341 results.

((“EEG” OR “electroencephalography” OR “electroencephalogram” OR “MEG” OR “magnetoencephalography”) AND (“resting-state”)) AND (“schizophrenia” OR “first episode of psychosis”)

EMBASE:887 results

(‘eeg’/exp OR ‘eeg’ OR ‘electroencephalography’/exp OR ‘electroencephalography’ OR ‘electroencephalogram’/exp OR ‘electroencephalogram’ OR ‘meg’ OR ‘magnetoencephalography’/exp OR ‘magnetoencephalography’) AND ‘resting-state’ AND (‘schizophrenia’/exp OR ‘schizophrenia’ OR ‘first episode of psychosis’)

PsycINFO (via EBSCO): 1209 results

(“EEG” OR “electroencephalography” OR “electroencephalogram” OR “MEG” OR “magnetoencephalography”) AND “resting-state” AND (“schizophrenia” OR “first episode of psychosis”)

CINAHL (via EBSCO): 54 results

(“EEG” OR “electroencephalography” OR “electroencephalogram” OR “MEG” OR “magnetoencephalography”) AND “resting-state” AND (“schizophrenia” OR “first episode of psychosis”)

LILACS: 1 result

(“EEG” OR “electroencephalography” OR “electroencephalogram” OR “MEG” OR “magnetoencephalography”) AND “resting-state” AND (“schizophrenia” OR “first episode of psychosis”)

the Cochrane Library: 0 result

(“EEG” OR “electroencephalography” OR “electroencephalogram” OR “MEG” OR “magnetoencephalography”) AND “resting-state” AND (“schizophrenia” OR “first episode of psychosis”)

Chinese database: 0 result

“精神分裂症” AND “静息态” AND (“伽马波” OR, γ) AND (“脑电图” OR “脑磁图”)
