# Supplementary material for: Resting-state gamma power in schizophrenia: a systematic review and meta-analysis
Source: Front Psychiatry. 2026 Jan 19;16:1731645. doi: 10.3389/fpsyt.2025.1731645 (PMC12862933; doi:10.3389/fpsyt.2025.1731645)
Supplement: Supplementary file 1 [file Table1.docx]

**Supplementary Material**

**Appendix.** Search Strategies

**Fig. S1.** Risk of bias of included studies

**Table S1.** GRADE evidence profile

**Table S2.** EEG-related features

**Appendix.** Search Strategies

MEDLINE (via PubMed): 341 results.

(("EEG" OR "electroencephalography" OR "electroencephalogram" OR "MEG" OR "magnetoencephalography") AND ("resting-state")) AND ("schizophrenia" OR "first episode of psychosis")

EMBASE：887 results

('eeg'/exp OR 'eeg' OR 'electroencephalography'/exp OR 'electroencephalography' OR 'electroencephalogram'/exp OR 'electroencephalogram' OR 'meg' OR 'magnetoencephalography'/exp OR 'magnetoencephalography') AND 'resting-state' AND ('schizophrenia'/exp OR 'schizophrenia' OR 'first episode of psychosis')

PsycINFO (via EBSCO): 1209 results

(“EEG” OR “electroencephalography” OR “electroencephalogram” OR “MEG” OR “magnetoencephalography”) AND “resting-state” AND (“schizophrenia” OR “first episode of psychosis”)

CINAHL (via EBSCO): 54 results

(“EEG” OR “electroencephalography” OR “electroencephalogram” OR “MEG” OR “magnetoencephalography”) AND “resting-state” AND (“schizophrenia” OR “first episode of psychosis”)

LILACS: 1 result

(“EEG” OR “electroencephalography” OR “electroencephalogram” OR “MEG” OR “magnetoencephalography”) AND “resting-state” AND (“schizophrenia” OR “first episode of psychosis”)

the Cochrane Library: 0 result

(“EEG” OR “electroencephalography” OR “electroencephalogram” OR “MEG” OR “magnetoencephalography”) AND “resting-state” AND (“schizophrenia” OR “first episode of psychosis”)

Chinese database: 0 result

“精神分裂症” AND “静息态” AND (“伽马波” OR, γ) AND (“脑电图” OR “脑磁图”)

**Fig. S1.** Risk of bias of included studies


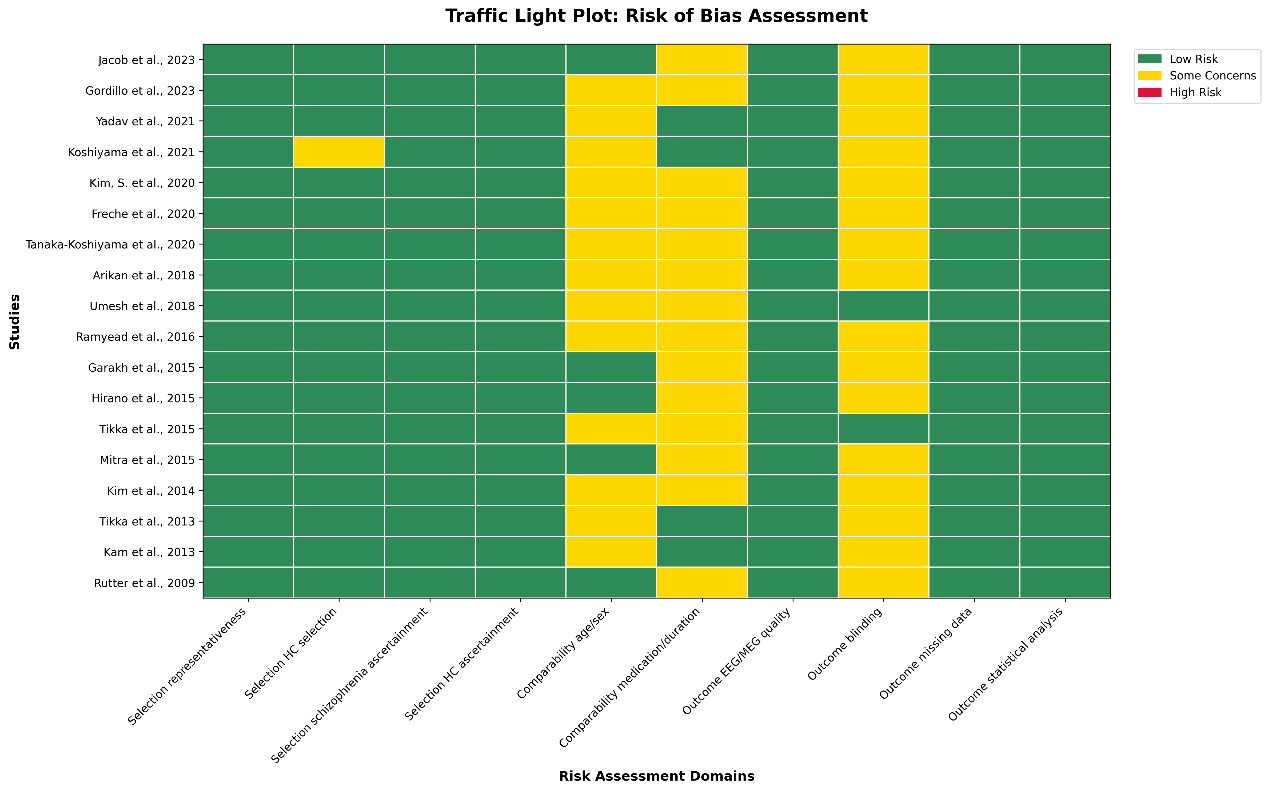


# Table S1. GRADE evidence profile

| Outcome | No. of studies | Certainty of evidence (GRADE) | Downgrading domains |
| --- | --- | --- | --- |
| Whole-brain gamma power (SZ vs HC) | 11 | Moderate | Risk of bias (moderate), Inconsistency (I²=78.2%), Reporting bias (trim-and-fill) |
| Left frontal gamma power | 8 | Low | Imprecision (95% CI crosses null), Few studies |
| Right frontal gamma power | 7 | Moderate | Inconsistency reduced (I² lower), moderate effect size, small sample sizes |
| Left temporal gamma power | 5 | Low | Imprecision, small N, CI includes null |
| Right temporal gamma power | 5 | Moderate | Moderate effect size but limited studies, CI near null |
| Left parietal gamma power | 6 | Low | Imprecision, inconsistency across studies |
| Right parietal gamma power | 5 | Low | Imprecision, CI wide, small number of studies |
| Left occipital gamma power | 5 | Low | Imprecision, small N, CI near null |
| Right occipital gamma power | 5 | Low | Imprecision, small N, CI near null |
| DMN regions (mPFC, PCC) | 4 | Low | Imprecision, very few studies (n=4), inconsistency, potential indirectness |

**Table S2.** EEG-related features

| Author | Recording time | Sampling rate | Filtering | Artifact rejection | Epochs | Data normalization | Source location |
| --- | --- | --- | --- | --- | --- | --- | --- |
|  |  |  |  |  |  |  |  |
| Rutter et al. 2009 | 4 min | 600 Hz | 0.61–150 Hz, 60 Hz notch | First and last 10s rejected | Na | Constant noise estimate; absolute/relative power use: Na | Synthetic aperture magnetometry (SAM); constant noise estimation normalization |
| Kam, Julia W Y et al.2013 | 3 min | 1000 Hz | High pass 0.5 Hz and 60 Hz notch | Exclusion of activity >100 V; algorithm for eye movement and blink removal; ICA | 2.048 s | Mean absolute power for each frequency band, logarithmic transformation | Na |
| S.K.Tikka et al.2013 | 10 min | 512 Hz | 30–100 Hz, 50 Hz notch | Visual inspection | Na | Recomputing with common average reference; Log transformation; use of absolute power | Na |
| Kim et al. 2014 | 150s | 1001 Hz | 0.1–200 Hz | Manual removal based on visual inspection | 2.56s | No; use of absolute power | sLORETA |
| S.Mitra et al.2015 | 3 min | 512 Hz | 0.1–120 Hz, 50 Hz notch | Visual inspection | 30s | Recomputing with common average reference; use of absolute power | Na |
| Ramyead, A. et al. 2015 | 20 min | 250 Hz | 1 Hz, 50 Hz notch | Visual inspection + ICA | 2s | Na; use of absolute power | eLORETA, statistical nonparametric mapping |
| S.K.Tikka et al.2015 | 10 min | 512 Hz | 30–100 Hz, 50 Hz notch | Visual inspection | Na | Recomputing with common average reference; Log transformation; use of absolute power | Na |
| Hirano, Yoji et al. 2015 | Na | 512 Hz | 0.1–100 Hz | Exclusion of activity>200 microV and variation >90 microvolt, ICA | 1s | Na, use of absolute power | Single epoch source dipole; BESA |
| Z. Garakh et al. 2015 | 100s | Na | 70 Hz | custom designed multiple-source eye correction method, visual inspection | 10–15s | Spectral power logarithmic; Transformation, use of relative power | Na |
| Umesh, S. et al.2018 | 10 min | 512 Hz | 0–120 Hz | Visual inspection | Na | Log transformation, application of Fisher Z, use of absolute power | Na |
| Arikan et al. 2018 | 3 min | 500 Hz | 0.15–70 Hz | Visual inspection | Na | Na; use of absolute power | Na |
| Tanaka-Koshiyama et al.2020 | 328s | 1000 Hz | 0.5–100 Hz | Visual inspection + EEGLAB plugin “clean raw data” + ICA | Na | Na | Equivalent current dipole with fieldtrip function |
| Freche, D. et al.2020 | 260 ± 60s | 1024 Hz | 1–150 Hz, 50 Hz notch | Visual inspection + ICA; elimination of initial and final epochs | Na | Data normalized using the quartile-based coefficient of variation; use of relative power | Na |
| Kim, S. et al.2020 | 5 min | 1000 Hz | 1–100 Hz | Visual inspection | 2s | Use of relative power | Depth-weighted minimum L2 norm estimator |
| S. Yadav et al.2021 | 10 min | Na | 0.1–120 Hz | Visual inspection | 60s | Log transformation; use of absolute power | Na |
| Koshiyama, D. et al. 2021 | 3 min | 1000 Hz | 0.5–100 Hz | EEGLAB plugin “clean raw data” including artefact subspace reconstruction | Na | Na | Equivalent current dipole using fieldtrip function |
| Dario Gordillo et al.2023 | 5 min | 2048 Hz | 0.1–100 Hz; 50 Hz notch | Visual inspection + ICA | 2s | Log transformation; use of absolute power | Na |
| Jacob, M. S. et al. 2023 | 6 min | Na | 1–200 Hz, 50 Hz notch | ICA; exclusion of variations > ±100 µV | 2s | Na | Na |

LORETA low-resolution tomography analysis
